# The Psychology of Eating

**DOI:** 10.3389/fpsyg.2013.00215

**Published:** 2013-04-25

**Authors:** Adrian Meule, Claus Vögele

**Affiliations:** ^1^Department of Psychology I, University of WürzburgWürzburg, Germany; ^2^Research Group “Self-Regulation and Health,” Research Unit INSIDE, Université du LuxembourgWalferdange, Luxembourg; ^3^Research Group on Health Psychology, University of LeuvenLeuven, Belgium

People engage in eating behavior as a matter of survival, normally every day. That is, one has to make choices about what to eat, when, and how much. In contrast to our ancestors, however, whose primary task was to seek out any food that would provide energy and nutrients, those choices have become more difficult nowadays. In western or westernized societies in particular, food is abundant, cheap, and available in a great variety. Moreover, eating is a fundamentally rewarding behavior, and is thus intrinsically linked to mood and emotions (Vögele and Gibson, 2010).

Because of this, we felt that the creation of a new specialty section about determinants and consequences of eating behavior and mechanisms of its modulation is warranted. The aim of *Frontiers in Eating Behavior* is to build knowledge for the understanding of eating behavior by bringing together academics with different expertise, e.g., researchers investigating basic processes related to eating behavior, clinical researchers examining the psychological, physiological, and nutritional aspects of eating disorders and practitioners, such as clinical psychologists, physicians, and other health care professionals. As yet, this new specialty section is the first and only periodical in open access publishing with a breadth of scope on eating behavior embracing various methodologies and study populations.

When humans are energy deficient, a complex interplay of physiological processes signals the brain that food should be consumed, i.e., an individual feels hungry. When enough food has been consumed, these processes signal that consumption should be terminated, i.e., an individual feels satiated or sated (Benelam, 2009). Nevertheless, this homeostatic regulation of eating is steadily challenged and overridden by the omnipresence of food and food-related cues. That is, eating can be triggered even in the absence of hunger or extended beyond satiation (Lowe and Butryn, 2007). Numerous factors are known that determine or guide eating behavior in an automatic and implicit fashion (Cohen and Farley, 2008). For instance, eating may be initiated or prolonged by the presence of others, i.e., is influenced by social factors (Herman and Polivy, 2004). Food choices and consumption are also strongly influenced by environmental factors, e.g., advertising, packaging, portion sizes, lighting, and many more (Stroebele and De Castro, 2004; Cohen and Babey, 2012). As a consequence, constant monitoring and self-regulation of eating is necessary in order to eat healthily, i.e., to provide the body both qualitatively and quantitatively with the right nutrients. At the same time, eating healthily also means to be able to enjoy the rewarding aspects of food without falling prey to a loss of control over eating.

Many individuals are able to do this successfully, yet some exhibit over-regulation of eating behavior resulting in underweight and malnutrition. Cases of anorexia nervosa have been known for a long time (Bemporad, 1996). On the other side of the extreme, permanent failures of self-regulation may result in overweight and obesity. As with anorexia, obesity is an age-old health condition (Haslam, 2011), but its prevalence has dramatically increased in the second half of the twentieth century (Stroebe, 2008). While prevalence rates in western countries appear to stabilize, rates of severe obesity continue to grow (Bessesen, 2008; Yanovski and Yanovski, 2011) and newly industrialized countries seem to catch up (Finucane et al., 2011).

In most cases, obesity is the result of poor dietary habits – rather than compulsive eating binges – which contribute to a modest average daily excess of energy intake over energy expenditure (Rogers, 2011). Some individuals, however, show regular binge eating which is defined as consuming large amounts of food over a discrete period of time with a sense of lack of control over eating, and which is associated with marked distress (American Psychiatric Association, 2013). Prevalence of binge eating disorder (BED) is increased in obese individuals, but not all patients with BED are necessarily obese. Furthermore, there are some individuals who engage in regular binge eating but use compensatory behaviors like vomiting to prevent weight gain. Hence, patients with bulimia nervosa (BN) are mostly normal-weight (Thompson, 2003).

Unlike anorexia and obesity, BED, and BN have been first described in the twentieth century but their research history yet encompasses several decades (Stunkard, 1959; Russell, 1979). Both eating disorders and obesity involve medical complications and are marked by psychological distress and co-morbid mental disorders (Thompson, 2003), of which mood and anxiety disorders are the most prevalent (Vögele and Gibson, 2010). Therefore, it is of no surprise that there are numerous scientific journals, which are devoted to publishing research on the etiology and treatment of those disorders.

In addition to eating disorders and obesity, there are a vast number of eating behaviors that deserve scientific scrutiny and discussion. For instance, there are some problematic eating behaviors that are not included in the current diagnostic manuals, but are continuously debated in terms of their clinical relevance (cf. Corsica and Pelchat, 2010; Vandereycken, 2011), e.g., night eating (Stunkard et al., 1955), orthorexia (Bratman and Knight, 2001), or food addiction (Randolph, 1956). Furthermore, there is an array of eating behaviors that do not reflect disordered eating *per se*, but nonetheless seem to be associated with occasional overeating and moderate overweight, e.g., restrained or emotional eating (Herman and Mack, 1975; Macht and Simons, 2011). Accordingly, it has been recognized that some eating behaviors can be mapped onto a continuum ranging from normal to disordered eating (e.g., Lowe et al., 1996).

Thus, it would appear to be inappropriate to focus on eating disorders and obesity as separate entities. We understand our mission for *Frontiers in Eating Behavior* to create a view on eating and its disorders on a continuum from healthy eating practices to disordered eating behaviors. Therefore, findings from basic research on eating behavior are important to understand disordered eating behavior. These findings may include research on how food and food-cues are processed in the brain, mechanisms underlying successful and unsuccessful self-regulation of eating, or social and environmental determinants of and individual differences in food choice and consumption. In addition, there is increasing evidence that eating behaviors, e.g., food preferences, are shaped by gene-environment interactions in early childhood. Nevertheless, the role of experience, or learning (e.g., classic conditioning, observational learning), is critical in the development of young children's eating behavior, which may well carry over into adulthood (Havermans, in press). Thus, a better understanding of the developmental aspects of eating behavior is essential to understand eating behavior in adulthood. On the other hand, results from clinical studies may advance our knowledge concerning non-clinical issues, which are relevant for most humans. For example, research on triggers of and treatment approaches for reducing binge eating may also be useful to increase dieting success or inducing healthier food choices in overweight individuals without eating disorders.

Thus, we encourage researchers to break away from isolated, categorical views on normal vs. disordered eating, homeostatic vs. hedonic concepts, or physiological vs. psychological mechanisms. We hope that *Frontiers in Eating Behavior* will increase the awareness of a more comprehensive view on eating behavior and advance our understanding of a behavior that is essential for our survival as individuals and as a species.
